# Refutation of traumatic insemination in the *Drosophila bipectinata* species complex

**DOI:** 10.1098/rsbl.2021.0625

**Published:** 2022-02-09

**Authors:** Michal Polak, Shane F. McEvey

**Affiliations:** ^1^ Department of Biological Sciences, University of Cincinnati, Cincinnati, OH 45221-0006, USA; ^2^ Australian Museum Research Institute, Australian Museum, 1 William Street, Sydney, NSW 2010, Australia

**Keywords:** *Drosophila bipectinata* species complex, traumatic insemination hypothesis, refutation, aedeagal lateral processes, genital claws, anchoring

## Abstract

Traumatic insemination (TI) is a rare reproductive behaviour characterized by the transfer of sperm to the female via puncture wounds inflicted across her body wall. Here, we challenge the claim made by Kamimura (Kamimura 2007 *Biol. Lett.* **3**, 401–404. (doi:10.1098/rsbl.2007.0192)) that males of species of the *Drosophila bipectinata* complex use a pair of claw-like processes (claws) to traumatically inseminate females: the claws are purported to puncture the female body wall and genital tract, and to inject sperm through the wounds into the lumen of her genital tract, bypassing the vaginal opening. This supposed case of TI is widely cited and featured in prominent subject reviews. We examined high-resolution scanning electron micrographs of the claws and failed to discover any obvious ‘groove’ for sperm transport. We demonstrated that sperm occurred in the female reproductive tract as a single-integrated unit, inconsistent with the claim that sperm are injected via paired processes. Laser ablation of the sharp terminal ends of the claws failed to inhibit insemination. We showed that the aedeagus in the complex delivers sperm through the vaginal opening, as in other *Drosophila*. The results refute the claim of TI in the *Drosophila bipectinata* species complex.

## 1. Introduction

Traumatic insemination (TI) is a form of mating behaviour during which males deploy specialized ‘devices', such as spines and stylets, to puncture the female body wall and transfer sperm through the wound(s) they inflict [1]. This extraordinary behaviour is distinguished from other forms of ‘traumatic mating’, where only non-sperm components of the ejaculate, or no ejaculate at all, transfer to the female through the male-inflicted wounds [1–4]. Though rare, TI has arisen independently in a number of animal groups [3,5], and the evolutionary drivers of this unusual form of insemination are the focus of ongoing debate [1,5–9].

It has been claimed by Kamimura [10] that TI occurs in species of the *Drosophila bipectinata* complex, a small taxonomic grouping of four very similar species within the *ananassae* subgroup, of the *melanogaster* species group, that includes: *Drosophila bipectinata* Duda, 1923; *Drosophila parabipectinata* Bock, 1971; *Drosophila malerkotliana* Parshad and Paika, 1965; and *Drosophila pseudoananassae* Bock, 1971 [11]. Specifically, males of this complex are purported by Kamimura to use a pair of claw-like phallic structures to pierce both the female body wall and reproductive tract, and to inject sperm into the lumen of her reproductive tract through the wounds. If true, then TI in the complex would be an astonishing evolutionary innovation within the genus, family and order.

In *Drosophila*, the ‘typical’ route of sperm transfer, storage and use is well documented in *Drosophila melanogaster* [12]: the ejaculate, comprising sperm and seminal plasma, passes via the tip of the aedeagus into the female bursa (uterus) through her gonopore (vaginal opening) [13–16]. The ejaculate, immediately after transfer to the female, exists in this and other species as a single sperm mass readily visualized by teasing open her reproductive tract and releasing it [15,17,18].

**Figure 1.**
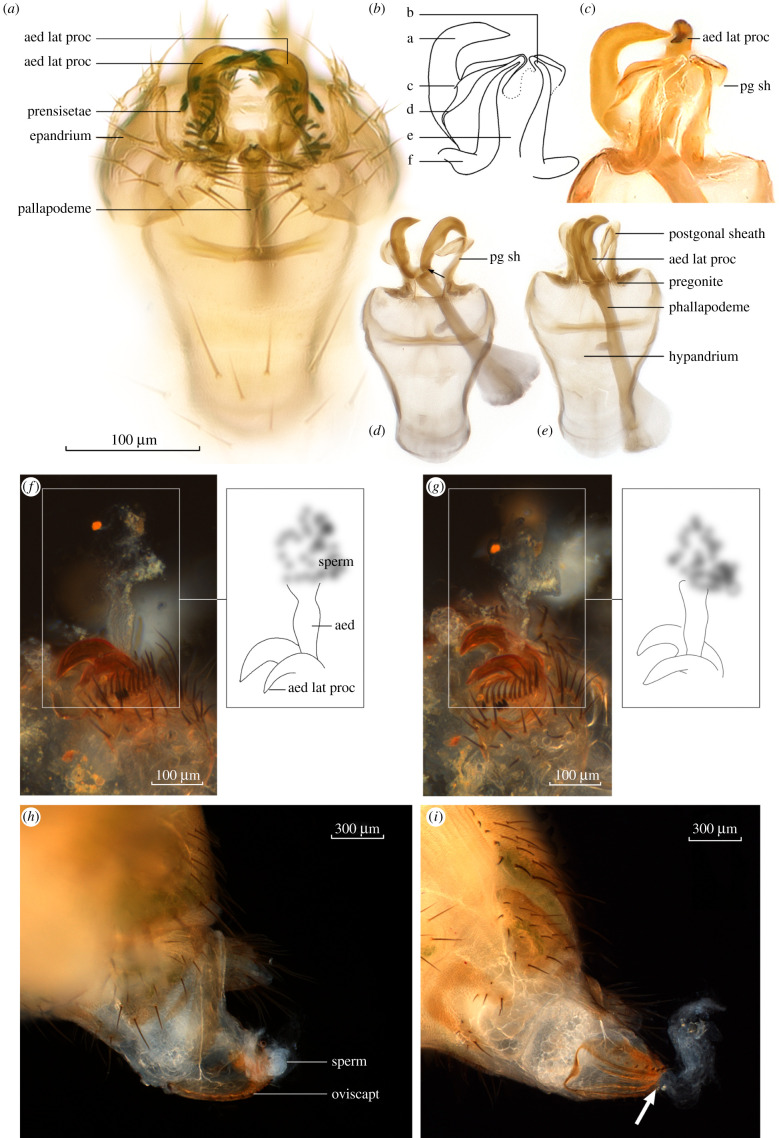
Light microscope images of reproductive structures of the *bipectinata* complex. (*a*) Hypandrium inside male body. (*b*,*c*) Drawing and digital image of posterior end of hypandrium, showing the aedeagal lateral processes (a, aed lat proc, claws) and the postgonal sheath (e, pg sh). Ridges and thickened processes (b, c, d, f) of the sheath are evident. (*d*,*e*) Aedeagal lateral processes and postgonal sheath in two phallapodeme orientations; articulation of the aedeagal lateral process with phallapodeme arrowed in (*d*). (*f*,*g*) Extruded aedeagus of a male *D. bipectinata*, arising from between the bases of the claws, showing sperm emanating from its tip. (*h*,*i*) Sperm seeping from the female gonopore (sperm teased further out arrowed in (*i*)).

In Kamimura's study, a laser scan micrograph showing two areas of coloration adjacent to the tips of the basal processes was claimed to demonstrate TI (Fig. 1c(ii) in [10]); the following is the relevant excerpt: ‘TI clearly occurs in the *bipectinata* complex, as the basal processes pierce the pockets during copulation and sperm is ejaculated through the wounds but not through the genital orifice…’ [10]. We were sceptical about this conclusion, as no evidence for the passage of sperm via such a route was actually provided. The following statement likewise gave us pause, as no direct evidence for it was presented either: ‘The basal processes of this group have a groove on the dorsal surface which may transport semen’ [10, p. 404].

Here, we challenge the claim that TI occurs in the *bipectinata* complex. Our results provide observational and direct experimental evidence contradicting it: the evidence supports sperm transfer occurring via the route described for other *Drosophila*, that is, from the aedeagus into the reproductive tract via the female gonopore. Recently, Rice *et al*. [19] showed that the claws have a distinct developmental origin, deriving from the lateral portions of the central primordium of the phallus observable during pupal metamorphosis. Since most cells of the central primordium normally give rise to the aedeagus, the term *aedeagal lateral processes* for the claw-like structures was proposed [19], a term we adopt here and use interchangeably with ‘claws’. Historically, the claws were thought to be arms of a bifid aedeagus [11,20–23] and were termed ‘basal processes’ by Kamimura [10].

We examined scanning electron micrographs (SEMs) of the claws at varying orientations and magnifications to search for a possible conduit for sperm—namely, Kamimura's purported ‘groove’. We then addressed two additional predictions. If insemination occurs traumatically via the paired claws, we would expect the sperm mass to occur as two discernible units within the female reproductive tract immediately after and/or during mating. To this end, we examined the sperm masses extracted from the female reproductive tract both immediately after the terminus of uninterrupted, full length (*ca* 10.5 min) copulations, and after pairs were interrupted 6–8 min after the onset of coupling, that is, during sperm transfer. Reproductive structures of both sexes were also examined after interrupted copulation, which led us to image the functional aedeagus and to identify the path of sperm passage to the female. As a final test, we used laser surgical ablation [24] to eliminate the pointed tips of the claws. If, as claimed by Kamimura, the claws serve to pierce the female's body wall and to transfer sperm to her reproductive tract, males with surgically ablated piercing devices should fail to transfer sperm.

## 2. Material and methods

### (a) Source and culturing of flies

*Drosophila bipectinata, D. parabipectinata* and *D. malerkotliana* cultures were established with field-caught flies; collection and culture methods are provided in the electronic supplementary material. Specimens from these cultures, used for morphological examination and imaging, are vouchered in the Australian Museum (K.380306, -07, -27).

### (b) Laser surgery

Laser surgical ablation is described in the electronic supplementary material. Pulses of laser light were used to ablate one-quarter to one-third of both claws of individual males—the ‘cut’ group. After surgery, males recovered in groups of 3–5 in food vials for at least 4 days until being paired with virgin females. ‘Uncut’ control males were treated identically, except that instead of cutting the claws, 2–4 large setae near the end of the abdomen were ablated [24].

### (c) Mating trials and dissections

Cut and uncut males were individually paired with a virgin female in 46 mating trials (detailed in the electronic supplementary material): the mating pairs in 42 trials (34 *D. bipectinata*, 8 *D. parabipectinata*) involved cut males, and males in four trials (2 *D. bipectinata*, 2 *D. parabipectinata*) were uncut controls. Immediately after decoupling, females were dissected and the sperm mass released into physiological saline to determine whether it occurred as a single unit or more than one unit. In separate trials (*n* = 12), copulation was interrupted 6–8 min after starting; immediately upon interruption, the female reproductive tract was dissected to visualize the sperm mass.

### (d) Imaging phallic structures

Genitalia were dissected from fresh and alcohol-preserved specimens and imaged using light and scanning electron microscopy (detailed in the electronic supplementary material). Membrane-clearing KOH was used for preparing the specimens in figure 1*a*–*e*, but not figure 1*f*–*i*. To search for possible sperm conduits along the claws, multiple SEMs were taken at different magnifications (350–3500 ×) and orientations. Genitalic preparations of 28 *D. parabipectinata*, 29 *D. bipectinata* and 3 *D. malerkotliana* were imaged using electron microscopy; a total of 166 SEMs of claws were examined and archived.

## 3. Results

### (a) Phallic architecture of the *bipectinata* species complex

**Figure 2.**
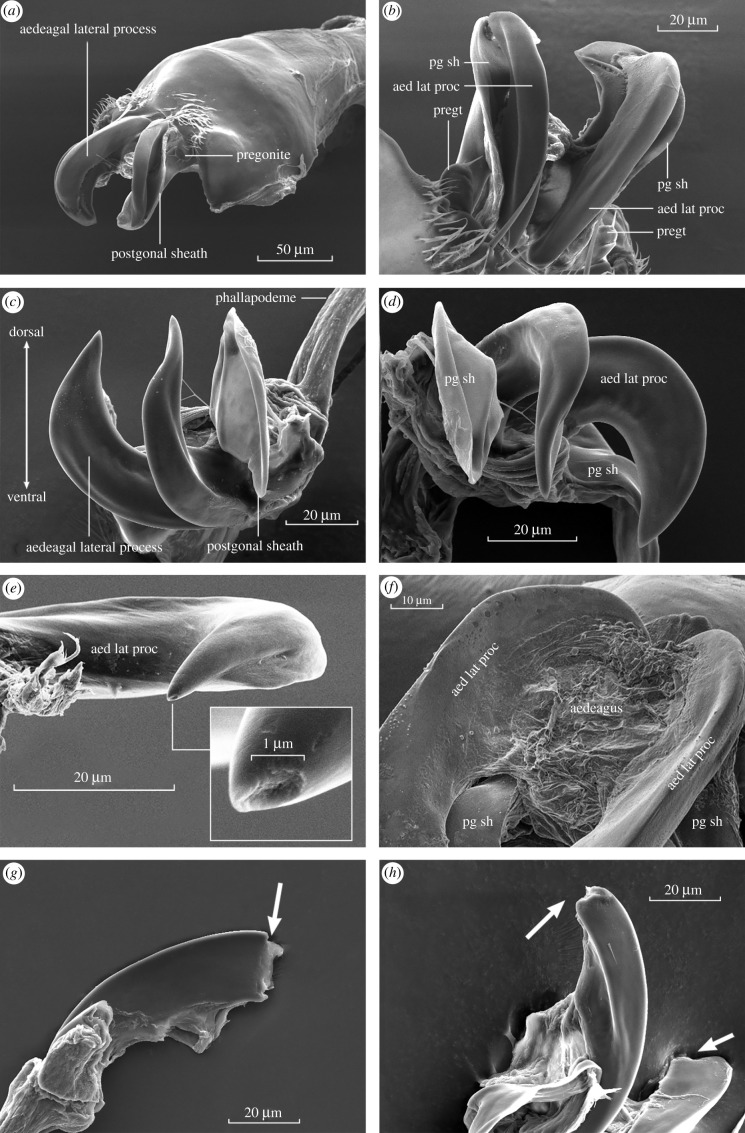
SEMs of the aedeagal lateral processes (aed lat proc) of the *bipectinata* complex. (*a*) Ventral surface of hypandrium with the pair of aedeagal lateral processes, cloaked in the postgonal sheath (pg sh). (*b*,*c*) Smooth, seamless ventral surfaces of the claws. Pregonite (pregt) with apical sensilla. (*d*) Ventral and lateral surfaces of the aedeagal lateral processes. Dehydration during scanning electron microscopy preparation likely accounts for the shallow medial depressions in (*c*) and (*d*). (*e*) Dorsal surface of an aedeagal lateral process with depression at its tip, possibly an abrasion. (*f*) Reticulated mat of tissue between the bases of the claws, likely to be the collapsed aedeagus. (*g*,*h*) Examples of the aedeagal lateral processes experimentally blunted (arrowed) using a surgical laser. Both tip-ablated processes visible in (*h*).

The phallic structures of all four species in the *bipectinata* complex are similar [13]; here, we describe the phallic architecture of *D. bipectinata* and *D. parabipectinata* as representing the complex. The aedeagal lateral processes (claws) are large, curved, apically pointed and bare (figures 1*c*,*d* and 2*a*–*e*), approximately 100 µm in length from base to tip, and they articulate with the apex of the phallapodeme (arrowed in figure 1*d*). The claws are bilaterally symmetrical and arise from the lateral portions (shoulders) (figure 1*a*,*d*,*e*), not the centre, of the phallapodeme apically. The membranous aedeagus arises centrally between the claws (figure 1*f*,*g*), but is not apparent after processing with KOH (figure 1*a*,*d*).

We see no connection between the base of the pregonites and the claws (figure 1*d*,*e*), which confirms that the claws are not ‘basal extensions’ of the pregonites evident in allied species of the *D. ananassae* complex [21,25]. The postgonal sheath (*sensu* [19]) folds and bends freely, and is bilaterally symmetrical, lobe-like and ‘cloaks’ the claws dorsally (figure 2*a*–*d*). When viewed via light microscopy, the postgonal sheath is largely membranous and translucent (figure 1*c*), with hardened outer ridges and leaf-like structure connected near the base of each claw (figure 1*b*–*e*).

### (b) The traumatic insemination hypothesis

We could identify no channel, groove or fold, medially, laterally, dorsally or ventrally on the claws that could function as a conduit for sperm (figure 2). In some preparations, we observed the tip of one or both claws to have an irregular depression or lesion (typically ≤ 1 µm in diameter), which could, owing to the irregular (torn) edges, be the result of abrasion (figure 2*e*).

When females were dissected at copulation termination, the sperm within the bursa invariably occurred as a single mass (electronic supplementary material, figure S1A). Out of a total of 12 matings interrupted at 6–8 min after the start of mating, 11 also invariably produced a single sperm mass within the female reproductive tract (electronic supplementary material, figure S1B). In three of these 11 cases, the sperm mass was small and appeared irregular in shape, amorphous, not smoothly oval or rounded, but nevertheless unquestionably as a single undifferentiated unit. In one interrupted mating, there was no ejaculate transfer at all.

The aedeagus was discovered when anaesthetized copulating pairs were gently pulled apart while submerged in saline solution*.* This organ in *D. bipectinata* is translucent, membranous and pliable, and it appears to have a textured (scaly) surface (figure 1*f*). Sperm were readily identified emanating from its tip (figure 1*f*,*g*) and could be gently drawn further out using a minuten pin. The aedeagus arises from between the bases of the aedeagal lateral processes (figure 1*f*,*g*)—in our SEMs, a reticulated mat of tissue between the bases of the claws could be discerned (figure 2*f*), which we interpret to be the collapsed aedeagus. In the female of the separated pair, sperm was observed seeping from the gonopore (the orifice of her reproductive tract through which eggs also exit) (figure 1*h*), and likewise, could be drawn further out of the gonopore (arrowed figure 1*i*).

Of the 30 total copulations achieved by cut males (figure 2*g*,*h*), 22 (73%) resulted in sperm transfer to the bursa as a single mass (electronic supplementary material, table S1 and figure S1C,D). Among the eight cut males that copulated but failed to transfer sperm to the female bursa, two remained fastened to the female in an end-to-end position after dismounting. In one case, ejaculate seeped out from between the pair and remained attached to the male's terminalia after the pair separated (electronic supplementary material, figure S2). This viscous, whitish mass contained sperm, indicating that it was leaked ejaculate, and that sexual union was incomplete.

## 4. Discussion

In their authoritative review of copulatory wounding, Lange *et al*. [1] listed a set of criteria for establishing the existence of traumatic mating in a given species. Here, building upon this work and that of Tatarnic *et al*. [5], we suggest that evidence for TI should minimally include (i) a specific wounding structure(s) that demonstrably breaches the female body wall; (ii) physical features of said structure(s), such as a canal, lumen, groove and/or pore, for the transfer and delivery of spermatozoa; and (iii) the transfer of spermatozoa across the female body wall. Several studies have demonstrated TI by fulfilling these criteria (e.g. [9,26–28]), a textbook example of which occurs in bed bugs (Cimicidae) ([26,27,29]; and see electronic supplementary material, figure S3).

By contrast, we contend that none of these criteria was convincingly met by Kamimura [10]. In the first place, whereas Kamimura claimed that integumental piercing is achieved by the claws, there was no direct evidence presented for physical penetration of the female body wall (nor the genital tract for that matter). The second criterion (the functional morphology of the organ) was not fulfilled either, as convincing visual evidence for a structure that could guide and transfer sperm across the female body wall was also not provided, and according to the present investigation, does not exist (and see below). Finally, although Kamimura claimed that ‘sperm is ejaculated through the wounds but not through the genital orifice’ [10, p. 404], the evidence he presented—areas of pink coloration in laser scan micrographs—we regard as unconvincing since the presence of sperm within the pink ‘clouds’ was not confirmed, let alone sperm transfer via the claws to the reproductive tract.

Our results refute the hypothesis of TI in the *bipectinata* species complex, and therefore in *Drosophila*, and for that matter in Diptera as far as we know. Sperm transfer in the *bipectinata* complex occurs as in *D. melanogaster*, from the male aedeagus into the female reproductive tract via her gonopore. We examined SEMs of the dorsal, lateral and ventral surfaces of the claws in *D. parabipectinata*, *D. bipectinata* and *D. malerkotliana* and could identify no obvious ‘groove’ for sperm transport. We next tested the prediction that immediately after and/or during mating, the ejaculatory mass within the female should be discernible as two units. The prediction failed, as sperm invariably occurred as a single mass, comporting with previous work [30]. We also tested the experimental prediction that after surgically eliminating the sharp terminal ends of the claws, insemination would be inhibited. This prediction also failed, as a majority of males (73%) without these sharp ends successfully inseminated females, and in all these cases, the sperm occurred as a single mass within the female reproductive tract.

Another decisive blow to the TI hypothesis is that we unambiguously imaged the functional aedeagus in the *D. bipectinata* complex. Pulling apart mating pairs of *D. bipectinata* clearly revealed sperm releasing simultaneously from the tip of a hitherto unknown aedeagus and the counterpart female gonopore, thus identifying the route of gamete transfer between the sexes. The aedeagus is membranous and becomes translucent in pre-dissection KOH treatment and readily collapses upon itself during scanning electron microscopy preparation, suggesting why it has been difficult to discern in previous studies.

The likely functions of the claws that we can discern are at least twofold, both of which are mechanical in nature and likely to be generating inter-locus sexual conflict [6]. One is that the claws serve to assist the male in achieving copulation by facilitating the grasping of the female and opening or orienting her gonopore—ability to ‘coerce’ previously inseminated females to mate may be an especially important determinant of male post-copulatory fitness [31,32]. A second potential (non-mutually exclusive) function is that they serve to anchor the genitalia and maintain intimate connection during copulation, thus facilitating the eversion of the aedeagus through the female gonopore and ejaculate transfer. A key observation in this regard is the seepage of ejaculate from between a mating pair involving a cut male (electronic supplementary material, figure S2), suggesting that claw removal resulted in the backflow of ejaculate, consistent with the anchoring hypothesis. We emphasize that additional experiments, which are beyond the scope of the present study, are needed to satisfactorily characterize the function of these remarkable structures now that we have demonstrated that they do not serve to traumatically inseminate females. Here, our primary objective was to address the TI hypothesis, and our results have led us to reject the assertion that TI occurs in the *Drosophila bipectinata* species complex.

## Data Availability

Data are available on Mendeley Data [33] and in the electronic supplementary material.

## References

[1] Lange R, Reinhardt K, Michiels NK, Anthes N. 2013 Functions, diversity, and evolution of traumatic mating. Biol. Rev. **88**, 585-601. (10.1111/brv.12018) 23347274

[2] Blanckenhorn WU, Hosken DJ, Martin OY, Reim C, Teuschl Y, Ward PI. 2002 The costs of copulating in the dung fly *Sepsis cynipsea*. Behav. Ecol. **13**, 353-358. (10.1093/beheco/13.3.353)

[3] Reinhardt K, Anthes N, Lange R. 2015 Copulatory wounding and traumatic insemination. Cold Spring Harb. Perspect. Biol. **7**, a017582. (10.1101/cshperspect.a017582) 25877218 PMC4448625

[4] Siva-Jothy MT. 2009 Reproductive immunity. In Insect infection and immunity: ecology, evolution and mechanisms (eds J Rolff, S Reynolds), pp. 241-251. Oxford, UK: Oxford University Press.

[5] Tatarnic NJ, Cassis G, Siva-Jothy MT. 2014 Traumatic insemination in terrestrial arthropods. Annu. Rev. Entomol. **59**, 245-261. (10.1146/annurev-ento-011613-162111) 24160423

[6] Arnqvist G, Rowe L. 2005 Sexual conflict. Princeton, NJ: Princeton University Press.

[7] Brand JN, Harmon LJ, Schaerer L. 2022 Frequent origins of traumatic insemination involve convergent shifts in sperm and genital morphology. Evol. Lett. (10.1002/evl3.268) PMC8802240 35127138

[8] Dougherty LR, van Lieshout E, McNamara KB, Moschilla JA, Arnqvist G, Simmons LW. 2017 Sexual conflict and correlated evolution between male persistence and female resistance traits in the seed beetle *Callosobruchus maculatus*. Proc. R. Soc. B **284**, 20170132. (10.1098/rspb.2017.0132) PMC5454259 28539510

[9] Tatarnic NJ, Cassis G. 2010 Sexual coevolution in the traumatically inseminating plant bug genus *Coridromius*. J. Evol. Biol. **23**, 1321-1326. (10.1111/j.1420-9101.2010.01991.x) 20456571

[10] Kamimura Y. 2007 Twin intromittent organs of *Drosophila* for traumatic insemination. Biol. Lett. **3**, 401-404. (10.1098/rsbl.2007.0192) 17519186 PMC2391172

[11] Bock IR. 1971 Taxonomy of the *Drosophila bipectinata* complex. Univ. Texas Publ. no. 7103, pp. 273-280.

[12] Gromko MH, Gilbert GG, Richmond RC. 1984 Sperm transfer and use in the multiple mating system of *Drosophila*. In Sperm competition and the evolution of animal mating systems (ed. RL Smith), pp. 371-426. Orlando, FL: Academic Press.

[13] Bairati A. 1968 Structure and ultrastructure of the male reproductive system in *Drosophila melanogaster* Meigen. The genital duct and accessory glands. Monit. Zool. Ital. (N.S.) **2**, 105-182.

[14] Fowler GL. 1973 Some aspects of the reproductive biology of *Drosophila*: sperm transfer, sperm storage, and sperm utilization. In Advances in genetics, vol. 17 (ed. EW Caspari), pp. 293-360. New York, NY: Academic Press.

[15] Manier MK, Belote JM, Berben KS, Novikov D, Stuart WT, Pitnick S. 2010 Resolving mechanisms of competitive fertilization success in *Drosophila melanogaster*. Science **328**, 354-357. (10.1126/science.1187096) 20299550

[16] Mattei AL, Riccio ML, Avila FW, Wolfner MF. 2015 Integrated 3D view of postmating responses by the *Drosophila melanogaster* female reproductive tract, obtained by micro-computed tomography scanning. Proc. Natl Acad. Sci. USA **112**, 8475-8480. (10.1073/pnas.1505797112) 26041806 PMC4500220

[17] Pitnick S, Markow TA. 1994 Male gametic strategies: sperm size, testes size, and the allocation of ejaculate among successive mates by the sperm-limited fly *Drosophila pachea* and its relatives. Am. Nat. **143**, 785-819. (10.1086/285633)

[18] Polak M, Starmer WT, Barker JSF. 1998 A mating plug and male mate choice in *Drosophila hibisci* Bock. Anim. Behav. **56**, 919-926. (10.1006/anbe.1998.0850) 9790703

[19] Rice GR, David JR, Gompel N, Yassin A, Rebeiz M. In press. Resolving between novelty and homology in the rapidly evolving phallus of *Drosophila* J. Exp. Zool. B Mol. Dev. Evol. (10.1002/jez.b.23113) PMC10155935 34958528

[20] Okada T. 1954 Comparative morphology of the drosophilid flies. I Phallic organs of the *melanogaster* group. Kontyu **22**, 36-48.

[21] Bock IR, Wheeler MR. 1972 The *Drosophila* *melanogaster* species group. Univ. Texas Publ. no. 7213, pp. 1-102.

[22] Gupta JP. 1973 Comparative studies of male genital structures of hybrids and their parental species. Experientia **29**, 224-225. (10.1007/BF01945490)

[23] Parshad R, Paika IJ. 1964 Drosophilid survey of India. II. Taxonomy and cytology of the subgenus *Sophophora* (*Drosophila*). Res. Bull. Punjab Univ. **15**, 225-252.

[24] Polak M, Rashed A. 2010 Microscale laser surgery reveals adaptive function of male intromittent genitalia. Proc. R. Soc. B **277**, 1371-1376. (10.1098/rspb.2009.1720) PMC2871932 20053645

[25] McEvey SF, Schiffer M. 2015 New species in the *Drosophila ananassae* subgroup from northern Australia, New Guinea and the South Pacific (Diptera: Drosophilidae), with historical overview. Rec. Austral. Mus. **67**, 129-161. (10.3853/j.2201-4349.67.2015.1651)

[26] Carayon J. 1966 Traumatic insemination and the paragenital system. In Monograph of Cimicidae, vol. 7 (ed. RL Usinger), pp. 81-166. College Park, MD: Entomological Society of America.

[27] Davis NT. 1956 The morphology and functional anatomy of the male and female reproductive systems of *Cimex lectularius* L. (Heteroptera, Cimicidae). Ann. Entomol. Soc. Am. **49**, 466-493. (10.1093/aesa/49.5.466)

[28] Řezáč M. 2009 The spider *Harpactea sadistica*: co-evolution of traumatic insemination and complex female genital morphology in spiders. Proc. R. Soc. B **276**, 2697-2701. (10.1098/rspb.2009.0104) PMC2839943 19403531

[29] Usinger R (ed.). 1966 Monograph of the Cimicidae. Philadelphia, PA: Entomological Society of America.

[30] Tyler F, Haverkos S, Imm A, Polak M. 2020 Analysis of correlated responses in key ejaculatory traits to artificial selection on a diversifying secondary sexual trait. J. Insect Physiol. **133**, 104291. (10.1016/j.jinsphys.2021.104291) 34364848

[31] Polak M, Hurtado-Gonzales JL, Benoit JB, Hooker KJ, Tyler F. 2021 Positive genetic covariance between male sexual ornamentation and fertilizing capacity. Curr. Biol. **31**, 1547-1554. (10.1016/j.cub.2021.01.046) 33567290

[32] Singh A, Singh BN. 2014 Studies on remating behaviour in the *Drosophila bipectinata* species complex: evidence for sperm displacement. Curr. Sci. **107**, 511-515.

[33] Polak M, McEvey SF. 2021 Polak & McEvey: copulation duration data in *Drosophila*. Mendeley Data. (10.17632/88bw5k8zb9.2)

